# Surprisingly low compliance to local guidelines for risk factor based screening for gestational diabetes mellitus - A population-based study

**DOI:** 10.1186/1471-2393-9-53

**Published:** 2009-11-16

**Authors:** Margareta Persson, Anna Winkvist, Ingrid Mogren

**Affiliations:** 1 Department of clinical science, Obstetrics and gynecology, Umeå University, Umeå, Sweden; 2 Institution of medicine, Department of clinical nutrition, Sahlgrenska Academy, University of Gothenburg, Gothenburg, Sweden

## Abstract

**Background:**

Screening for gestational diabetes mellitus (GDM) is routine during pregnancy in many countries in the world. The screening programs are either based on general screening offered to all pregnant women or risk factor based screening stipulated in local clinical guidelines. The aims of this study were to investigate: 1) the compliance with local guidelines of screening for GDM and 2) the outcomes of pregnancy and birth in relation to risk factors of GDM and whether or not exposed to oral glucose tolerance test (OGTT).

**Methods:**

This study design was a population-based retrospective cross-sectional study of 822 women. A combination of questionnaire data and data collected from medical records was applied. Compliance to the local guidelines of risk factor based screening for GDM was examined and a comparison of outcomes of pregnancy and delivery in relation to risk factor groups for GDM was performed.

**Results:**

Of the 822 participants, 257 (31.3%) women fulfilled at least one criterion for being exposed to screening for GDM according to the local clinical guidelines. However, only 79 (30.7%) of these women were actually exposed to OGTT and of those correctly exposed for screening, seven women were diagnosed with GDM. Women developing risk factors for GDM during pregnancy had a substantially increased risk of giving birth to an infant with macrosomia.

**Conclusion:**

Surprisingly low compliance with the local clinical guidelines for screening for GDM during pregnancy was found. Furthermore, the prevalence of the risk factors of GDM in our study was almost doubled compared to previous Swedish studies. Pregnant women developing risk factors of GDM during pregnancy were found to be at substantially increased risk of giving birth to an infant with macrosomia. There is a need of actions improving compliance to the local guidelines.

## Background

Gestational diabetes mellitus (GDM) is defined as carbohydrate intolerance that is initiated or detected during pregnancy [1]. GDM is associated with other pregnancy complications and is an indicator of future development of diabetes mellitus type 2 [2]. In Sweden, the prevalence of GDM is reported in 1.2 - 2.3% of the pregnant women [3-6]. Since the 1990s, Sweden has officially adopted the European recommendation of selective screening for GDM [7]. However, a Swedish audit of all local guidelines for screening, diagnostics and treatment for GDM reveals no national unified guidelines [8].

Risk factors for GDM may be categorized in maternal or pregnancy-related factors. The risk factors include obesity, multiparity, high maternal age, family history of diabetes mellitus and a previous delivery with a macrosomic infant [2]. Elevated levels of fasting plasma glucose [9], repeated glucosuria and elevated levels of random plasma glucose during pregnancy are pregnancy-related risk factors for GDM [10] as well as accelerated foetal growth and polyhydramniosis [11].

Few studies have examined compliance with local guidelines of screening for GDM. In a Thai study, the compliance to local guidelines for GDM screening was 78% of the pregnant women [12]. Further, compliance of local guidelines for GDM in Australia was 95.3% [13], while the compliance rate in France was 80% [14]. Even though the compliance rate is high in these studies, not all qualified women underwent screening as recommended in the local guidelines. Hence some women with GDM may go undetected. Untreated GDM or impaired glucose tolerance (IGT) seem to increase the risk of adverse outcomes such as macrosomia, and cesarean sections [15,16], as well as an increased risk of preterm delivery [16].

The aims of this study were to investigate:

1. The compliance with local guidelines of screening for GDM.

2. The outcomes of pregnancy and birth in relation to risk factors of GDM and whether or not exposed to oral glucose tolerance test (OGTT).

## Method

The study design was a population-based, retrospective, cross-sectional study combining data from a questionnaire with data of medical records. The Regional Ethical Review Board, University of Umeå, Sweden, has approved the study (Dnr 05-020M).

### Procedure

**Figure 1:**
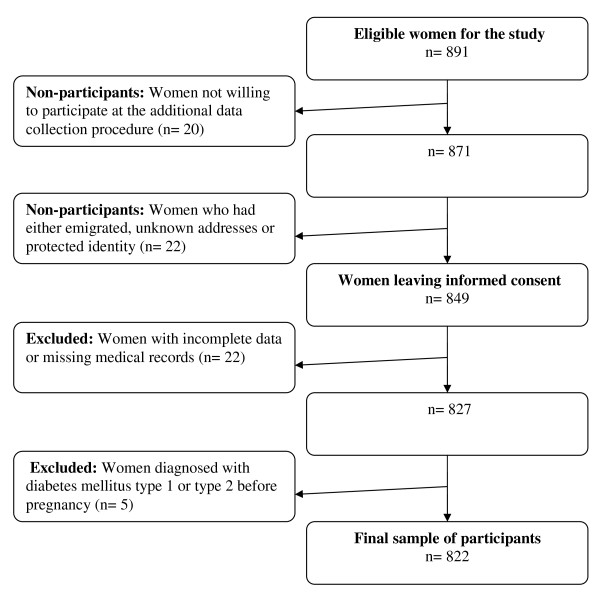
**The selection of the sample of participants**.

The current study is an extension of a previous questionnaire study investigating low back and pelvic girdle pain during pregnancy. The initial recruitment procedure has been reported elsewhere in detail [17-19]. Data were gathered between January 1, 2002 through April 30, 2002 from Umeå University Hospital (UUH) and Sunderby Hospital (SH), both located in the northern part of Sweden. The initial data set for this study consisted of 891 women. Inclusion criteria for participation were delivery after 23 gestational weeks and proficiency in the Swedish language. All women participating in the initial study were contacted through a letter sent by mail with information of the extension of the study. It was explained in the letter that they could withdraw from further participation by returning a prepaid envelope with a note saying that they declined further participation. After the informed consent of the participants, comprehensive information on the outcomes of pregnancy and childbirth was collected from medical records and added to the previously collected questionnaire data. The selection of the final sample is presented in Figure 1. The participants in this study consisted of 822 women, constituting 73.8% of the total eligible population of 1114 women who had delivered within the two hospitals during the same period.

### Questionnaire and medical data

The questionnaire covered different aspects of gynaecologic and obstetric history of the participating women as well as questions concerning life style before and during pregnancy. Parts of these data have been presented in relation to low back and pelvic girdle pain [17-19]. From each medical record, 75 items presenting maternal characteristics and pregnancy outcomes were collected and added to the questionnaire data. These items included the presence of risk factors according to the local guidelines of GDM screening, medical outcomes of pregnancy and birth, and medical diagnosis using International Classification of Diseases (ICD) version 10.

### Local guidelines of screening for GDM

The local guidelines obtained for both catchment areas, addresses the risk factors which indicates screening for GDM. Compilation of local guidelines for each catchment area is presented in Table 1. The majority of the criteria were identical in the catchment areas. According to the guidelines, the OGTT should be performed in early pregnancy in women with prior GDM and repeated at 28 gestational weeks if the first OGTT were normal. Other risk factors indicated that OGTT should be performed at 28 gestational weeks. Blood glucose levels were measured independent of last meal of the pregnant woman at 4 times regularly during pregnancy in the SH catchment area and at 5-6 times regularly during pregnancy in the UUH area. When a high blood glucose value would be detected, the OGTT should be performed immediately independent of gestational age.

**Table 1:** Criteria indicating oral glucose tolerance test (OGTT) screening for gestational diabetes mellitus (GDM) and cut-off threshold value for GDM diagnosis according to the local guidelines 2002 within the two catchment areas.

Criteria indicating OGTT screening for GDM Cut-off threshold at OGTT indicating GDM diagnosis	Umeå University Hospital UUH)	Sunderby Hospital (SH)
**Risk factors in medical history**		
Family history of diabetes (parents and siblings)	Yes	Yes
Previous pregnancy with GDM	Yes	Yes
Previous child with birth weight ≥4500 g	Yes	Yes
Maternal body weight before pregnancy (kg)	≥90	≥90
Body Mass Index, BMI (kg/m^2^)	No	≥33
**Risk factors developed during pregnancy**		
Randomly controlled blood glucose level at any antenatal visit during pregnancy (mmol/l)	≥8.0	≥7.1
Accelerated fetal growth	Yes	Yes
Polyhydramniosis	Yes	Yes
**Cut-off threshold at 75 g OGTT indicating GDM diagnosis**		
2 hours blood glucose value (mmol/l)	≥9.0	≥8.9

### Definitions

#### Maternal measures

*Hypertension *was defined as blood pressure ≥140/90 mm Hg. The hypertensive group consisted of the subgroups of the ICD-10 codes, i.e. ICD O10.0 (pre-existing essential hypertension complicating pregnancy, childbirth and the puerperium) and ICD O13 (gestational [pregnancy-induced] hypertension without significant proteinuria).

*Preeclampsia *was defined as a blood pressure ≥140/90 mm Hg with proteinuria of ≥1+ on a dipstick. The ICD-10 codes of O14 (gestational [pregnancy-induced] hypertension with significant proteinuria with all subcodes) and O15 (eclampsia) were included as preeclampsia.

*Preterm birth *was defined as gestational age < 37 completed weeks. Gestational age was determined by ultrasound for almost all women.

*Oral glucose tolerance test (OGTT) *was used to screen for GDM. All women received 75 g glucose after ≥8 hours of fasting. Blood glucose level was measured after 2 hours.

*Gestational diabetes mellitus *(GDM) was defined as the ICD-10 code O24.4 diabetes mellitus arising in pregnancy. The risk factors indicating OGTT screening as well as the cut-off threshold at OGTT identifying GDM diagnosis in the study regions are presented in Table 1.

*Accelerated foetal growth *was defined when at least two values of the symfys-fundus measures exceeded >2 standard deviations (SD) recorded in the antenatal medical records. Furthermore, the ICD-10 code O36.6 (maternal care for excessive foetal growth) was also used for identifying cases with accelerated foetal growth. *Polyhydramniosis *was defined using the ICD-10 code O40 polyhydramniosis.

*Birth experience *was the women's overall estimation of the experience of birth. The women were asked to give a comprehensive assessment of their experience by marking a Visual Analogue Scale (VAS) from 0 to 10, where anchors where verbally defined as 0 = most negative and 10 = most positive.

#### Neonatal measures

*Macrosomia*, was defined as ICD-10 code of P08.0 (exceptionally large baby or a recorded birth weight ≥4500 g).

*Jaundice *was defined as ICD-code P59.0 (neonatal jaundice associated with preterm delivery) and P59.9 (neonatal jaundice, unspecified).

*Hypoglycemia *consisted of the ICD-10 codes P70.0 (syndrome of infant of mother with gestational diabetes) and P70.4 (other neonatal hypoglycemia).

### Non-participants

Some available data from the questionnaires allowed comparisons between participants and non-participants. The non-participants (n = 69) did not differ significantly from participants (n = 822) concerning maternal age, parity, educational level, gestational age at birth, mode of delivery, or birth experience. A comprehensive description of non-participants in the initial data collection has been reported elsewhere [18].

### Categorization of participants

**Figure 2:**
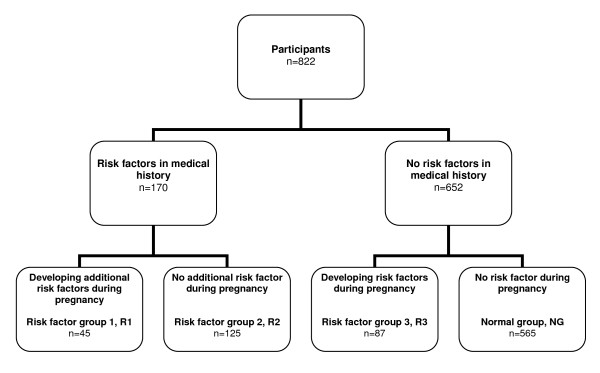
**Categorization of participants depending on presence of risk factors for GDM**.

To compare the characteristics and outcomes of women with and without risk factors of GDM, the women were categorized into groups based on presence of risk factors as defined in the local guidelines (Figure 2). Firstly, all women were dichotomized with respect the presence or absence of GDM risk factors in their medical history. Secondly, within these two groups, all women were dichotomized with respect to developing GDM risk factors or not during pregnancy. Based on these categorizations, three groups of women with risk factors were identified.

1. Risk factor group1, R1: women with risk factors in medical history and additional risk factors developing during pregnancy.

2. Risk factor group 2, R2: women with risk factors in their medical history.

3. Risk factor group 3, R3: women with risk factors developing during pregnancy.

A fourth group of women with absence of any risk factors in their medical history or during pregnancy (normal group, NG) was also identified.

### Statistical analysis

Statistical analyses were performed using the statistical software package (SPSS, version 16.0). For analyses of categorical variables, the Chi square-test was performed. For small samples, the Fischer's exact test was applied. For normally distributed continuous variables, the Student's t-test and analysis of variance [ANOVA] were performed. The Bonferroni procedure was used to control for multiple testing. For variables with a skewed distribution, the Kruskal-Wallis and Mann-Whitney tests were applied. The level of significance was set at *p *= 0.05.

Univariate and stepwise multiple logistic regression analyses were used to investigate the association between exposures before and during pregnancy and outcomes of pregnancy and birth. The analyses of risk factors were conducted in two steps. All exposure variables were tested one by one in separate, univariate analysis. In a second step, all statistically significant variables in the univariate analysis were tested using multivariate logistic regression analysis. Significant variables were entered in a stepwise manner. Results from the final model are presented as odds ratios (ORs) with confidence interval (CI) 95%.

Three models were tested in the analysis. Model 1 consisted of the group of women fulfilling the criteria of OGTT being correctly exposed to the test and the normal group of women with absence of GDM risk factors in their medical history and during pregnancy. Model 2 was based on the group of women fulfilling the criteria of OGTT and not being exposed to the test, and the normal group of women with the absence of GDM risk factors in their medical history and during pregnancy. Finally, the third model consisted of the women who fulfilled the criteria, but were not exposed to the test and the women fulfilling the criteria for OGTT and correctly exposed to the test.

### Validity of the data

The internal and external validity of the questionnaire has been discussed elsewhere. Twenty-five women completed an identical questionnaire two to three weeks after the first questionnaire and the consistency between the two sets was high, with total agreement in majority of questions [17-19].

To test the internal validity of data from the medical records, a control of 50 randomly selected medical journals was performed. Of the 3 750 items controlled, 5 items (0.1%) of incorrect data excerption were found and corrected.

## Results

### Risk factors and OGTT

At the first antenatal visit, at least one risk factor for GDM in the medical history as stipulated in the local guidelines was reported by 170 (20.7%) women. There were no significant differences in prevalence of risk factors in medical history between the two catchment areas. Among the women with risk factors in their medical history diabetes mellitus in family was the most frequent risk factor (61%), followed by maternal body weight of 90 kg or more and/or BMI ≥33 (38%), previous infant with birth weight of 4500 g or more (14%) and previous pregnancy with GDM (1%). As presented in Additional file 1, women with risk factors in their medical history reported more problems related to life style and had lower educational level. Most women were married or cohabiting with their partner. The women with risk factors in their medical history described their work as being less intellectually stimulating (71.3% vs. 81.5%, p = 0.034). No differences were found regarding other aspects of the work such as the work being sedentary or psychologically demanding. Furthermore, there were no significant differences concerning the evaluation of the relationship with spouse/partner and satisfaction with sex-life.

Risk factors developing during pregnancy were reported in 132 pregnancies (15.9%). Additional file 1 presents maternal characteristics of pregnant women with risk factors for GDM in their medical history and/or developing during pregnancy and pregnant women with no risk factors for GDM in their medical history. Of the 132 women, 45 women (33.6%) already had at least one risk factor for GDM in their medical history. The most frequent risk factor developed during pregnancy was accelerated foetal growth (59%), followed by random high blood glucose value (42%) and polyhydramniosis (1%). The women developing risk factors during pregnancy were significantly heavier, 74.6 (± 16.9) kg vs. 65.5 (± 11.2) kg, p < 0.001 and correspondingly presenting a higher BMI 27.8 (± 5.9) vs. 24.3 (± 4.0), p < 0.001 compared to women not developing risk factors of GDM during pregnancy. No other significant differences were observed between the women developing risk factors during pregnancy and those who did not.

The 257 women included in the categories R1, R2 and R3 (ie. 31.3% of the total sample) fulfilled criteria for performing OGTT as stipulated in the local clinical guidelines. However, only 84 women were actually exposed to OGTT according to their medical records (Additional file 1). Five of the 84 women had no indication of OGTT presented in their medical records (although performed), leaving 79 out of 257 (30.7%) women correctly exposed to OGTT. Seven women (8.8%) of the women correctly exposed of OGTT were diagnosed with GDM. Women in R1 underwent an OGTT significantly more often compared to the women in R2 or R3. Obesity was the most frequent risk factor (in the medical history) in combination with accelerated foetal growth during pregnancy in the R1. Family history of diabetes mellitus was the most prevalent risk factor for R2. Among the women in R3, accelerated foetal growth was the most prevalent risk factor. As presented in Additional file 1, the women included in the R1 were significantly more obese than the women included in the other risk factor groups, even when a body weight ≥90 kg was one of the risk factors.

### Outcomes of pregnancy and birth in relation to risk factor groups

The outcomes of pregnancy and birth in relation to risk factor groups are presented in Table 2. The mean birth weight was significantly higher in R1 and R3 as well as the mean placenta weight in relation to NG. The correlation coefficient between birth weight and placental weight (subjects with twin pregnancies and/or preterm birth were excluded from the analysis) was r^2 ^= 0.41, p < 0.001. Similar results for the four subgroups were r^2 ^(R1) = 0. 51, r^2 ^(R2) = 0. 42, r^2 ^(R3) = 0.39 and r^2^(NG) = 0.34. There were no significant differences between the subgroups concerning mode of delivery, preterm delivery, induction of labour, haemorrhage, Apgar score, pH of the umbilical cord blood, and proportion of children referred to neonatal intensive care unit. Nor were there any significant differences concerning jaundice or hypoglycaemia of the infant observed. Women developing risk factors during pregnancy (R1 and R3) had significantly more often been exposed to induced labour (19% vs. 12%, p = 0.037) compared to women with no risk factors during pregnancy (R2 and NG). The birth experience did not show significant differences between the subgroups, although women with risk factors in their medical history (R1 and R2) expressed significantly more negative birth experience scores; 7.6 (± 2.4) vs. 8.0 (± 2.0), p = 0.026 compared to women with no risk factors in their medical history (R3 and NG).

**Table 2:** Statistical comparisons of outcomes of pregnancy and birth for women in study groups

	*Risk factors for GDM in medical history* *(n = 170)*		*No risk factors for GDM in medical history* *(n = 652)*		*P value*
	
	Risk factor group 1, R1^†^ (n = 45)	Risk factor group 2, R2^†^ (n = 125)	Risk factor group 3, R3^†^ (n = 87)	Normal group, NG^†^ (n = 565)	
**Maternal weight gain in kg during pregnancy**, mean (± SD)	11.0^1 ^(± 7,3)	12.4 (± 5,5)	13.8^2 ^(± 5,7)	12.8 (± 4,8)	0.028
**Highest systolic blood pressure (mm Hg)**, median (25 - 75 quartiles)	140^2 ^(125-147)	130^2 ^(120-140)	130^2 ^(120-135)	125 ^2 ^(120-133)	< 0.001
**Highest diastolic blood pressure (mm Hg)**, median (25 - 75 quartiles)	87 ^3 ^(80-95)	80 (75-85)	80 (75-85)	80 (73-84)	< 0.001
**Highest random blood glucose value (mmol/l)**, median (25 - 75 quartiles)	6.7 ^4 ^(5,7-8,0)	5.7 (5,3-6,2)	6.7 ^4 ^(5,6-7,7)	5.6 (5,1-6,2)	< 0.001
**Proteinurea during pregnancy**	18 (40%)	25 (20%)	18 (21%)	112 (20%)	0.017
**Preeclampsia, ICD-10 codes O14+O15**	5 (11%)	6 (5%)	4 (5%)	22 (4%)	0.172
**Gestational weeks at birth**, mean (± SD)	39.1 (± 1.89)	39.0 (± 2.22)	39.1 (± 2.24)	39.0 (± 2.16)	0.964
**Birth weight of child (g)**, mean (± SD)	3958^5 ^(± 673)	3570^5 ^(± 709)	3773^5 ^(± 703)	3469 (± 600)	< 0.001
**Weight of placenta (g)**, median (25-75 quartiles)	692^6 ^(609-800)	632^6 ^(520-705)	670^6 ^(600-790)	585^6 ^(509-660)	< 0.001
Birth experience*, mean (± SD)	7.36 (± 2,3)	7.69 (± 2,5)	7.74 (± 2,4)	8.04 (± 1.9)	0.064

^† ^R1: Women with risk factors for GDM in medical history and developing additional risk factors during pregnancy, R2: Women with risk factors for GDM in medical history and not developing additional risk factors during pregnancy, R3: Women with no risk factors for GDM in medical history and developing additional risk factors during pregnancy and NG: Women with no risk factors for GDM in medical history nor during pregnancy

* The women's overall experience of birth was expressed on a scale where the anchors where verbally defined as 1 = very negative and 10 = very positive.

^1^/R1 vs. R3 p-value = 0.022; ^2^/R1 vs. NG p < 0.001, R1 vs. R2 p = 0.004, R1 vs. R3 p = 0.003, R2 vs. NG p = 0.032, R2 vs. R3 p = ns, R3 vs. NG p = 0.022; ^3^/R1 vs. R2, R3 and NG p < 0.001; ^4^/R1 and R3 vs. R 2 and NG p < 0.001; ^5^/R1 and R3 vs. NG p < 0.001, R1 vs. R2 p = 0.003; ^6^/R1 vs. NG p < 0.001, R1 vs. R2 p = 0.004, R1 vs. R3 ns, R2 vs. NG p = 0.004, R2 vs. R3 p = 0.04, R3 vs. NG p < 0.001

### Risk of macrosomic infant

In univariate logistic regression analysis, delivery of macrosomic infant was significantly associated with the determinants: multiparity (≥3 children), education lower than university level, previous infant with macrosomia, maternal bodyweight ≥90 kg, accelerated foetal growth, being correctly exposed to OGTT and weight gain of 16 kg or more during pregnancy. In the stepwise multiple logistic regression model, the determinants previous infant with macrosomia (OR 17.77, 95% CI 1.38-229.15) and maternal body weight ≥90 kg at entrance of pregnancy (OR 17.80, 95% CI 1.06-299.40) remained significant.

R1 demonstrated an increased risk of a macrosomic infant (OR 5.65, 95% CI 2.64-12.11) as did R3 (OR 3.48, 95% CI 1.27-9.57) in relation to NG. Adjusting for multiparity (≥3 children), education less than university level, maternal body weigh ≥90 kg at entrance of pregnancy and weight gain of ≥16 kg, R1 had a substantially increased risk (OR 9.33, 95% CI 2.65-32.87) of giving birth to a macrosomic infant as did women in R3 (OR 12.96, 95% CI 1.33-126.63) compared to NG.

### Outcomes of pregnancy and birth for women correctly exposed to OGTT and women fulfilling criteria of OGTT but not exposed to OGTT

R2 and R3 had an increased risk of non-exposure to OGTT in relation to R1 (R2: OR 7.01, 95% CI 3.30-14.89 and R3: OR 8.49, 95% CI 3.75-19.21). As presented in Additional files 2 and 3, women fulfilling criteria of OGTT and correctly exposed to OGTT and the women fulfilling criteria but not exposed, gave birth to infants with macrosomia significantly more frequent compared to NG. In the stepwise multiple regression, the risk of an infant with macrosomia was more than 4-folded for the women correctly exposed to OGTT and more than 5-folded for the women not exposed to OGTT. As presented in Additional file 4, in the univariate analysis, women fulfilling criteria, however not exposed to OGTT presented decreased risk of obesity, diastolic blood pressure of ≥140 mm Hg, proteinuria and macrosomia. Further, a more negative birth experience was found among these women compared to women correctly exposed to OGTT. However, in the stepwise multiple regression analysis, only the outcome of a decreased risk of developing proteinuria during pregnancy remained significant. Adjusting for all risk factors of GDM (as defined in the local guidelines), women with the lowest education level had an increased risk (OR 6.19, 95% CI 1.58-24.27) of not being correctly exposed to OGTT when fulfilling the criteria for performing the test in relation to women with university education.

## Discussion

The findings in this study within a Swedish context indicate surprisingly low compliance with the local guidelines for screening pregnant women for GDM. Close to one third of all pregnant women presented at least one risk factor of GDM in their medical history or a risk factor developing during pregnancy. Furthermore, women developing risk factors for GDM during pregnancy were at increased risk of giving birth to an infant with macrosomia.

In the 1990s in Australia, half of the pregnant women were not screened for GDM during pregnancy despite the recommendation of OGTT screening for all pregnant women [20]. In the last couple of years, the reported compliance ranged from 78% - 92.1% [12-14]. These studies demonstrate considerably higher prevalence of performed screening for GDM compared to our study. The current study did not investigate the possible causes to the low compliance to the local guidelines of screening for GDM. The women were commonly referred to the clinical laboratory at the local health care centre or the nearby hospital for the performance of OGTT. This situation might have negatively influenced the patient adherence in relation to OGTT performed within the midwifery service. Another possible explanation could be that some pregnant women might have refused to undergo OGTT. Further, risk factors in the medical history might not have been correctly observed by the midwives. The medical history was routinely requested at the first antenatal visit, but by the time of arrangement for the OGTT, this initial information of risk factors might have been overlooked.

Obesity is one of the risk factors indicating screening for GDM, and obesity might be a sensitive topic to approach for midwives. It has been reported that nurses are aware of the obesity stigma, which implies communication tactics, such as softening the terms and avoiding the term 'obesity' due to its negative connotations. This strategy has been applied to deal with the sensitivity and maintain good relationship [21]. Moreover, by increasing the knowledge and competence of midwives in addressing sensitive issues, improved communication skills might be achieved, which may be beneficial for the pregnant women.

The prevalence of the risk factors for GDM in the medical history in a Swedish population has previously been estimated to reach 15.8%. This estimation is based on the risk factors family history of diabetes mellitus, maternal body weight ≥90 kg, prior delivery of an infant of ≥4500 g (ie. macrosomia) or prior GDM [3]. In our study, the prevalence of risk factors among the pregnant population was higher; 20.7% of the pregnant women had at least one of the risk factors for GDM in their medical history. Adding the risk factors developing during pregnancy, almost one third (31.3%) of all pregnant women had risk factors indicating risk for developing GDM. Despite the recommendations of OGTT stipulated in the local guidelines, merely one in three pregnant women with risk factors underwent an OGTT. In the present study, the local guidelines indicated a body weight of 90 kg as a cut-off value for screening because of obesity. This cut-off level may be questioned since a short woman with a body weight below the cut-off level may be obese, hence at risk for GDM. This was the case in our material as 6% of the participants with absence of risk factors in the medical history had a BMI corresponding to obesity, but did not fulfill the criteria of the risk factor body weight in the local guidelines. The risk of development of GDM during pregnancy is doubled at BMI 25-29.9 and six-folded at BMI ≥30 compared to pregnant women with normal BMI [22]. A national audit of all local guidelines for GDM screening in Sweden shows that a cut-off value of 90 kg for body weight as an indicator for obesity was applied in 28% of the local guidelines [8].

There is an ongoing international debate on the value of GDM screening programs. There are some suggestions that screening for GDM in all pregnant women may improve maternal and foetal outcomes [23,24], and some evidence that treatment of GDM after 24 gestational weeks improves maternal and foetal outcomes [24]. Others propose temporary suspension of the screening and treatment for GDM or elevating the cut-off threshold indicating a GDM diagnosis until a clear benefit of an intervention has been presented [25]. In Sweden, there is no national consensus addressing the screening for GDM, thus there are regions with general screening as well as regions with selective screening based on risk factors [8]. In a recent publication from the Hyperglycemia and Adverse Pregnancy Outcome (HAPO) study, the authors conclude that there is no specific threshold at which risks for adverse outcomes increase. Increased birth weights are observed even when maternal blood glucose level is below the values indicating GDM [26]. The same relation may be the case in our study as R1 and R3 demonstrated significantly higher random glucose levels and further, an increased risk of delivery of a macrosomic infant.

Strong associations with subsequent development of diabetes mellitus type 2 (DM2) after GDM have been reported. A systematic review reports that the cumulative incidence after the index pregnancy with GDM ranges between 2.6% to over 70% depending on the follow-up period [27]. Recently, the increased risk of DM2 after a previous GDM was estimated to more than 7-folded in relation to women with normal blood glucose levels during pregnancy [28]. Scandinavian studies show that 35 - 41% of women with previous GDM develop DM2 within 10 - 15 years [29,30]. Regarding these aspects, the low compliance to the local guidelines in our study must be considered disadvantageous. Close to one third of the pregnant women in the current study was characterized by having at least one risk factor for developing GDM during pregnancy, a situation that highlights an increasing problem within public health of women. Evidently, not all women with risk factors for GDM will develop GDM during pregnancy; however the midwives identify women with a number of health problems who might benefit from health interventions that could improve women's future health.

### Methodological considerations

In Sweden, almost all deliveries take place in hospitals. Since less than 0.5/1000 are planned home births [31], the population-based design with data collection before discharge from hospital after childbirth accurately reflect the population. The validity of the data from the questionnaires has been extensively discussed elsewhere [17-19]. The current study combined data from medical records with self-reported results from a questionnaire. Furthermore, the data were collected within two separate regions in the northern part of Sweden.

A retrospective data collection procedure may induce recall-bias. However, the accuracy of perinatal information has been reported as very good even four to six years after childbirth [32]. The data from the women were collected within the first days after delivery and birth in our study, which most probably will account for accurate memories and statements by the participants. However, the accuracy of midwives' and physicians' documentation may be questioned. The documentation in medical records by midwives and physicians show that midwives have higher accuracy of recorded medical conditions, pregnancy complications, intrapartum and postpartum events than physicians in a study in the USA [33]. A validation of the excerpted data in the current study was performed showing that only a few items were incorrect.

In order to improve the compliance to the local guidelines, the OGTTs could preferably be performed within the midwifery service. Furthermore, there is a need of national consensus of the design of a screening program for GDM. Further studies should address the issue of compliance with local guidelines to verify whether these findings are general. Future studies may address midwives' attitudes toward obesity and women with low educational levels. Furthermore, investigation of whether interventions regarding diet and blood glucose levels may influence the prevalence of macrosomia among women developing risk factors during pregnancy should be conducted.

## Conclusion

In conclusion, a surprisingly low compliance with the local guidelines of screening for GDM was found. Furthermore, the prevalence of the risk factors for GDM in our study was almost doubled compared to previous Swedish studies. Pregnant women developing risk factors of GDM during pregnancy were found to be at increased risk of giving birth to an infant with macrosomia.

## Competing interests

The authors declare that they have no competing interests.

## Authors' contributions

MP participated in the design of the study, carried out the excerption of data from medical records, performed the statistical analysis in collaboration with IM, and drafted the manuscript. AW participated in the design of the study, and has contributed to the analysis and final manuscript. IM participated in the design of the study, and supervised the statistical analysis and contributed to the manuscript. All authors read and approved the final manuscript.

## Pre-publication history

The pre-publication history for this paper can be accessed here:

http://www.biomedcentral.com/1471-2393/9/53/prepub

## Supplementary Material

Additional file 1 **Statistical comparisons of maternal characteristics of study groups**. The data provided represent the statistical comparison of maternal characteristics of the study groups. Click here for file

Additional file 2 **Odds ratios (ORs) and their 95% confidence intervals (CIs) for outcomes of pregnancy and birth for women correctly exposed to OGTT in relation to women not fulfilling criteria for OGTT in univariate and stepwise multivariate logistic regression.** Numbers included in analyses are specified for each variable. The data provided represent the outcomes of pregnancy and birth for women correctly exposed to OGTT in relation to women not fulfilling criteria for OGTT. Click here for file

Additional file 3 **Odds ratios (ORs) and their 95% confidence intervals (CIs) for outcomes of pregnancy and birth for women fulfilling criteria for OGTT, however not exposed to OGTT in relation to women not fulfilling criteria for OGTT in univariate and stepwise multivariate logistic regression.** Numbers included in analyses are specified for each variable. The data provided represent the outcomes of pregnancy and birth for women fulfilling criteria for OGTT, however not exposed to OGTT in relation to women not fulfilling criteria for OGTT. Click here for file

Additional file 4 **Odds ratios (ORs) and their 95% confidence intervals (CIs) for outcomes of pregnancy and birth for women fulfilling criteria for OGTT, however not exposed to OGTT in relation to women correctly exposed to OGTT in univariate and stepwise multivariate logistic regression.** Numbers included in analyses are specified for each variable. The data provided represent the outcomes of pregnancy and birth for fulfilling criteria for OGTT, however not exposed to OGTT in relation to correctly exposed to OGTT women. Click here for file
