# Supplementary material for: Surprisingly low compliance to local guidelines for risk factor based screening for gestational diabetes mellitus - A population-based study
Source: BMC Pregnancy Childbirth. 2009 Nov 16;9:53. doi: 10.1186/1471-2393-9-53 (PMC2784436; doi:10.1186/1471-2393-9-53)
Supplement: Additional file 1 — Statistical comparisons of maternal characteristics of study groups. The data provided represent the statistical comparison of maternal characteristics of the study groups. [file 1471-2393-9-53-S1.DOC]

Additional file 1. Statistical comparisons of maternal characteristics of study groups.

|  | ***Riskfactors for GDM in*** ***medical history***  *( n =170)* | ***No riskfactors for GDM in medical history***  *(n =652)* | *P value* |  | ***Risk factors for GDM in medical history***  *(n =170)* | | ***No risk factors for GDM in medical history***  *(n =652)* | | *P value* |
| --- | --- | --- | --- | --- | --- | --- | --- | --- | --- |
|  |  |  |  |  | **Risk factor group 1, R1^†^**  (n=45) | **Risk factor group 2, R2^†^**  (n=125) | **Risk factor group 3, R3^†^**  (n=87) | **Normal group, NG^†^**  (n=565) |  |
|  |  |  |  |  |  |  |  |  |  |
| **Maternal age**, mean (± SD) | 29.7 (± 5.4) | 29.6 (± 4.7) | n.s |  | 29.7 (± 5.3) | 29.7 (± 5.4) | 29.5 (± 5.1) | 29.6 (± 4.7) | n.s |
|  |  |  |  |  |  |  |  |  |  |
| **Maternal age at birth of first child,** mean (± SD) | 25.2 (± 4.5) | 26.6 (± 4.6) | <0.001 |  | 24.8 (± 3.6)^1^ | 25.3 (± 4.8)^2^ | 26.5 (± 4.7) | 26.7 (± 4.6) | 0.003 |
|  |  |  |  |  |  |  |  |  |  |
| **Parity,** mean (± SD) | 2.2 (± 1.1) | 1.8 (± 0.9) | <0.001 |  | 2.2 (± 1.1) | 2.2 (± 1.1)^3^ | 1.8 (± 0.9)^3^ | 1.8 (± 0.9)^3^ | 0.001 |
|  |  |  |  |  |  |  |  |  |  |
| **Age at menarche,** mean (± SD) | 12.4 (± 1.5) | 12.9 (± 1.3) | <0.001 |  | 12.2 (± 1.3)^4^ | 12.5 (± 1.4)^5^ | 12.7 (± 1.2) | 12.9 (± 1.3) | 0.001 |
|  |  |  |  |  |  |  |  |  |  |
| **Highest educational level**  Compulsory school/ folk high school  High school  University | 20 (12%)  90 (54%)  58 (34%) | 30 (5%)  298 (46%)  317 (49%) | <0.001 |  | 4 (9%)  23 (54%)  16 (37%) | 16 (13%)^6^  67 (54%)  42 (34%) | 6 (7%)  41 (47%)  40 (46%) | 24 (4%)  257 (46%)  277 (50%) | 0.002 |
|  |  |  |  |  |  |  |  |  |  |
| **Smoking before pregnancy** | 46 (28%) | 111 (17%) | 0.002 |  | 14 (33%)^7^ | 32 (26%)^7^ | 17 (20%) | 94 (17%) | 0.014 |
|  |  |  |  |  |  |  |  |  |  |
| **Use of alcohol before pregnancy,** ≥once a week | 34 (21%) | 225 (36%) | 0.002 |  | 6 (14%)^8^ | 28 (23%)^8^ | 31 (36%) | 194 (36%) | 0.003 |
|  |  |  |  |  |  |  |  |  |  |
| **Ever been physically active on a regular basis** | 122 (73%) | 535 (83%) | 0.004 |  | 32 (73%)^9^ | 90 (73%)^9^ | 79 (92%)^9^ | 456 (84%) | 0.004 |
|  |  |  |  |  |  |  |  |  |  |
| **Satisfied with pre-pregnancy weight** | 58 (35%) | 402 (62%) | <0.001 |  | 9 (21%)^10^ | 49 (40%)^10^ | 46 (54%) | 356 (64%) | <0.001 |
|  |  |  |  |  |  |  |  |  |  |
| **Perceived problem with actual or previous overweight** | 87 (52%) | 139 (22%) | <0.001 |  | 33 (79%)^11^ | 54 (44%)^11^ | 25 (34%)^11^ | 111 (20%) | <0.001 |
|  |  |  |  |  |  |  |  |  |  |
| **Ever been on diet** | 114 (69%) | 319 (50%) | <0.001 |  | 37 (88%)^12^ | 77 (62%)^12^ | 51 (60%)^12^ | 268 (48%) | <0.001 |
|  |  |  |  |  |  |  |  |  |  |
| **Maternal body weight at first antenatal visit, kilograms,** median, (25 – 75 quartiles) | 77.5 (67.0-94.0) | 65.0 (59.0-72.0) | <0.001 |  | 93.0^13^  (83.0-93.0) | 74.0^13^  (64.5-89.5) | 70.0^13^  (62.5-77.0) | 64.0  (59.0-74.0) | <0.001 |

| **Maternal Body Mass Index (BMI) at first antenatal visit,** median,  (25- 75 quartiles) | 27.3 (24.1-33.35) | 23.4 (21.6-26.2) | <0.001 |  | 33.3^14^  (28.3-36.7) | 26.0^14^  (23.2-30.9) | 24.8^14^  (22.5-27.9) | 23.2  (21.5-25.8) | <0.001 |
| --- | --- | --- | --- | --- | --- | --- | --- | --- | --- |
|  |  |  |  |  |  |  |  |  |  |
| **Body Mass Index (BMI) groups ***  BMI < 18.5  BMI 18.5 – 24.99  BMI 25.0 - 29.99  BMI 30.0 – 34.99  BMI ≥ 35.0 | 2 (1%)  56 (33%)  47 (28%)  34 (20%)  31 (18%) | 11 (1%)  414 (64%)  187 (29%)  37 (6%)^#^  0 (0%) | <0.001 |  | 0 (0%)^15^  4 (9%)  15 (33%)  9 (20%)  17 (38%) | 2 (2%)^15^  52 (42%)  32 (26%)  25 (20%)  14 (11%) | 2 (3%)^15^  41 (48%)  32 (38%)  10 (12%)  0 (0%) | 9 (2%)  373 (66%)  155 (28%)  27 (5%)  0 (0%) | <0.001 |
|  |  |  |  |  |  |  |  |  |  |
| **Oral Glucose Tolerance Test (OGTT) accomplished**  (R1 – R3 fulfilling criteria for OGTT) |  |  |  |  | 31 (69%)^16^ | 30 (24%)^16^ | 18 (21%)^16^ | 5  (1%)^17^ | < 0.001 |
|  |  |  |  |  |  |  |  |  |  |
| **GDM diagnosis after OGTT** |  |  |  |  | 5 (11%)^18^ | 1 (0.8%) | 1 (1%) | 0 | <0.001 |
|  |  |  |  |  |  |  |  |  |  |

^†^ R1: Women with risk factors for GDM in medical history and developing additional risk factors during pregnancy, R2: Women with risk factors for GDM in medical history and not developing additional risk factors during pregnancy, R3: Women with no risk factors for GDM in medical history and developing additional risk factors during pregnancy and NG: Women with no risk factors for GDM in medical history nor during pregnancy

* BMI groups are defined according to WHO definitions where underweight is < 18.50 kg/m^2^ , normal weight 18.50 – 24.99 kg/m^2^, overweight 25.00 -29.99 kg/m^2^ , obesity 30.00 – 34.99 kg/m^2^ and extreme obesity ≥ 35.00 kg/m^2^

^#^ These women did not fulfil the criteria of body weight ≥ 90 kg or BMI ≥ 33 as recommended in the local guidelines; i.e. short women with overweight.

^1^ R1 vs. NG, p=0.057; ^2^ R2 vs. NG, p=0.022; ^3^ R2 vs. R3, p=0.036, R2 vs. NG, p=0.002; ^4^ R1 vs. NG, p=0.002;  ^5^ R2 vs. NG, p=0.014; ^6^R2 vs. NG, p<0.001; ^7^R1 vs. NG, p=0.009, R2 vs. NG, p=0.019; ^8^R1 vs. R3, p=0.008, R1 vs. NG, p=0.009, R2 vs. R3, p=0.041, R2 vs. NG, p=0.011; ^9^R1 vs. R3, p=0.003, R2 vs. R3, p=0.001, R2 vs. NG, p=0.038, R3 vs. NG, p=0.017; ^10^R1 vs. R2, p=0.034, R1 vs.R3, p<0.001, R1 vs. NG, p<0.001, R2 vs. R3, p=0.037, R2 vs. NG, p<0.001; ^11^R1 vs. R2, p<0.001, R1 vs. R3, p<0.001, R1 vs. NG, p<0.001, R2 vs. NG, p<0.001, R3 vs. NG, p=0.004; ^12^R1 vs. R2, p=0.002, R1 vs. R3, p=0.001, R1 vs. NG, p<0.001, R2 vs. NG, p=0.004, R3 vs. NG, p=0.038; ^13^R2 vs. R3 p=0.005, all others p<0.001; ^14^R2 vs. R3 p=0.02, all others p<0.001; ^15^R1 vs. R2, p<0.001, R1 vs. R3, p<0.001, R1 vs. NG, p=0.001, R2 vs. R3, p= 0.006, R2 vs. NG, p=0.001, R3 vs. NG, p=0.005; ^16^R2 vs. R3 n.s., all others p<0.001 ^17^OGTT performed although no medical reasons registered in medical records; ^18^R1 vs. R2, p=0.005, R1 vs.R3, p=0.009, R1 vs. NG, p<0.001
