# Supplementary material for: Surprisingly low compliance to local guidelines for risk factor based screening for gestational diabetes mellitus - A population-based study
Source: BMC Pregnancy Childbirth. 2009 Nov 16;9:53. doi: 10.1186/1471-2393-9-53 (PMC2784436; doi:10.1186/1471-2393-9-53)
Supplement: Additional file 3 — Odds ratios (ORs) and their 95% confidence intervals (CIs) for outcomes of pregnancy and birth for women fulfilling criteria for OGTT, however not exposed to OGTT in relation to women not fulfilling criteria for OGTT in univariate and stepwise multivariate logistic regression. Numbers included in analyses are specified for each variable. The data provided represent the outcomes of pregnancy and birth for women fulfilling criteria for OGTT, however not exposed to OGTT in relation to women not fulfilling criteria for OGTT. [file 1471-2393-9-53-S3.DOC]

Additional file 3. Odds ratios (ORs) and their 95% confidence intervals (CIs) for outcomes of pregnancy and birth for women fulfilling criteria for OGTT, however not exposed to OGTT in relation to women not fulfilling criteria for OGTT in univariate and stepwise multivariate logistic regression. Numbers included in analyses are specified for each variable.

|  | *Total number* | *Obesity BMI≥ 30^1^,*  *n (%)* | *Crude OR (95% CI)* | *Total number* | *Systolic blood pressure ≥ 140 ^2^, n (%)* | *Crude OR (95%CI)* | *Total number* | *Diastolic blood pressure ≥ 90 ^3^, n (%)* | *Crude OR (95%CI)* | *Total number* | *Proteinuria^4^,*  *n (%)* | *Crude OR (95%CI)* | *Total number* | *Birth weight ≥4500gr^5^,*  *n (%)*  *(n=737)*  *17 (3.0%)*  *16 (9.0%)* | *Crude OR (95% CI)* | *Total number* | *Less positive birth experience, ≤ 5.0 ^6^,*  *n (%)* | *Crude OR (95%CI)* |
| --- | --- | --- | --- | --- | --- | --- | --- | --- | --- | --- | --- | --- | --- | --- | --- | --- | --- | --- |
| Normal group, no indication of OGTT (N=565) | 406 | 27  (6.7%) | 1.0 | 560 | 85  (15.25) | 1.0 | 560 | 63  (11.2%) | 1.0 | 556 | 111  (20.0) | 1.0 | 559 | 17  (3.0%) | 1.0 | 482 | 43  (8.9%) | 1.0 |
| Fulfilling criteria for OGTT, not exposed to OGTT (N=178) | 120 | 43  (35.8%) | 7.84  (4.57-  13.49) | 178 | 47  (26.4%) | 2.00  (1.34-  3.01) | 178 | 29  (16.3%) | 1.54  (0.95-2.47) | 176 | 35  (19.9) | 1.0 | 178 | 16  (9.0) | 3.15  (1.56-  6.37) | 155 | 25  (16.1%) | 1.96  (1.15-  3.34) |
|  |  |  |  |  |  |  |  |  |  |  |  |  |  |  |  |  |  |  |
| **Model 2**  **Stepwise multiple regression*** |  |  | Adjusted OR  (95%CI) |  |  | Adjusted OR  (95%CI) |  |  | Adjusted OR  (95%CI) |  |  | Adjusted OR  (95%CI) |  |  | Adjusted OR  (95%CI) |  |  | Adjusted OR  (95%CI) |
| Normal group, no indication of OGTT  Fulfilling criteria for OGTT, not exposed to OGTT |  |  | 1.0  5.92  (3.20-  10.96) |  |  | 1.0  1.49  (0.69-  3.23) |  |  | 1.0  0.89  (0.35-2.28) |  |  | 1.0  0.72  (0.37-  1.41) |  |  | 1.0  5.30  (1.98-14.21) |  |  | 1.0  1.85  (0.92-  3.72) |

* = Last step of stepwise multiple logistic regression presented in table.

1) Weight dichotomised as normal body weight BMI ≤ 24.99 and obesity as BMI ≥ 30

2) Systolic blood pressure dichotomised as systolic blood pressure < 140 mm Hg and systolic blood pressure ≥ 140 mm Hg

3) Diastolic blood pressure dichotomised as diastolic blood pressure < 89 mm Hg and diastolic blood pressure ≥ 90 mm Hg

4) Proteinuria dichotomized as no prevalence of proteinuria and prevalence of proteinuria

5) Birth weight dichotomised as birth weight < 4500 g and birth weight ≥ 4500 g

6) Experience of birth dichotomized as ‘less positive experience’ < 5.0 and ‘positive birth experience’ ≥ 7.0.
