# Supplementary material for: Surprisingly low compliance to local guidelines for risk factor based screening for gestational diabetes mellitus - A population-based study
Source: BMC Pregnancy Childbirth. 2009 Nov 16;9:53. doi: 10.1186/1471-2393-9-53 (PMC2784436; doi:10.1186/1471-2393-9-53)
Supplement: Additional file 4 — Odds ratios (ORs) and their 95% confidence intervals (CIs) for outcomes of pregnancy and birth for women fulfilling criteria for OGTT, however not exposed to OGTT in relation to women correctly exposed to OGTT in univariate and stepwise multivariate logistic regression. Numbers included in analyses are specified for each variable. The data provided represent the outcomes of pregnancy and birth for fulfilling criteria for OGTT, however not exposed to OGTT in relation to correctly exposed to OGTT women. [file 1471-2393-9-53-S4.DOC]

|  | *Total number* | *Obesity BMI ≥ 30^1^,*  *n (%)* | *Crude OR (95% CI)* | *Total number* | *Systolic blood pressure ≥ 140 ^2^, n (%)* | *Crude OR (95%CI)* | *Total number* | *Diastolic blood pressure ≥ 90 ^3^, n (%)* | *Crude OR (95%CI)* | *Total number* | *Proteinuria^4^, n (%)* | *Crude OR (95%CI)* | *Total number* | *Birth weight ≥4500gr^5^,*  *n (%)*  *(n=737)*  *17 (3.0%)*  *16 (9.0%)* | *Crude OR (95% CI)* | *Total number* | *Less positive birth experience, ≤ 5.0 ^6^,*  *n (%)* | *Crude OR (95%CI)* |
| --- | --- | --- | --- | --- | --- | --- | --- | --- | --- | --- | --- | --- | --- | --- | --- | --- | --- | --- |
| Fulfilling criteria for OGTT, correctly exposed to OGTT (N=79) | 56 | 32  (57.1%) | 1.0 | 79 | 30  (38.0%) | 1.0 | 79 | 24  (30.4%) | 1.0 | 79 | 26  (32.9%) | 1.0 | 79 | 11  (13.9%) | 1.0 | 72 | 12  (16.7%) | 1.0 |
| Fulfilling criteria for OGTT, not exposed to OGTT (N=178) | 120 | 43  (35.8%) | 0.42  (0.22-  0.80) | 178 | 47  (26.4%) | 0.57  (0.33 -  1.03) | 178 | 29  (16.3%) | 0.45  (0.24 -0.83) | 176 | 35 (19.9%) | 0.51  (0.28 –  0.92) | 178 | 16  (9.0%) | 0.61  (0.27 –  1.38) | 155 | 25  (16.1%) | 0.96  (0.45 –  2.04) |
|  |  |  |  |  |  |  |  |  |  |  |  |  |  |  |  |  |  |  |
| **MODEL 3**  **Stepwise multiple regression *** |  |  | Adjusted OR (95%CI) |  |  | Adjusted OR (95%CI) |  |  | Adjusted OR (95%CI) |  |  | Adjusted OR (95%CI) |  |  | Adjusted OR (95%CI) |  |  | Adjusted OR (95%CI) |
| Fulfilling criteria for OGTT, correctly exposed to OGTT  Fulfilling criteria for OGTT, not exposed to OGTT |  |  | 1.0  0.49  (0.22 –  1.09) |  |  | 1.0  1.21  (0.41-  3.57) |  |  | 1.0  0.34  (0.11-1.05) |  |  | 1.0  0.43  (0.18-  0.99) |  |  | 1.0  1.08  (0.35-  3.37) |  |  | 1.0  0.65  (0.24-  1.73) |

Additional file 4. Odds ratios (ORs) and their 95% confidence intervals (CIs) for outcomes of pregnancy and birth for women fulfilling criteria for OGTT, however not exposed to OGTT in relation to women correctly exposed to OGTT in univariate and stepwise multivariate logistic regression. Numbers included in analyses are specified for each variable.

* = Last step of stepwise multiple logistic regression presented in table.

1) Weight dichotomised as normal body weight BMI ≤ 24.99 and obesity as BMI ≥ 30

2) Systolic blood pressure dichotomised as systolic blood pressure < 140 mm Hg and systolic blood pressure ≥ 140 mm Hg

3) Diastolic blood pressure dichtomised as diastolic blood pressure < 89 mm Hg and diastolic blood pressure ≥ 90 mm Hg

4) Proteinuria dichotomized as no prevalence of proteinuria and prevalence of proteinuria

5) Birth weight dichotomised as birth weight < 4500 g and birth weight ≥ 4500 g

6) Experience of birth dichotomized as ‘less positive experience’ < 5.0 and ‘positive birth experience’ ≥ 7.0
